# The Early Postnatal Life: A Dynamic Period in Thymic Epithelial Cell Differentiation

**DOI:** 10.3389/fimmu.2021.668528

**Published:** 2021-06-18

**Authors:** Ruben G. R. Pinheiro, Nuno L. Alves

**Affiliations:** ^1^ i3S – Instituto de Investigação e Inovação em Saúde, Universidade do Porto, Porto, Portugal; ^2^ IBMC – Instituto de Biologia Molecular e Celular, Universidade do Porto, Porto, Portugal; ^3^ Doctoral Program in Cell and Molecular Biology, Instituto de Ciências Biomédicas, Universidade do Porto, Porto, Portugal

**Keywords:** thymus, thymic epithelial cells, tolerance, early postnatal life, aging

## Abstract

The microenvironments formed by cortical (c) and medullary (m) thymic epithelial cells (TECs) play a non-redundant role in the generation of functionally diverse and self-tolerant T cells. The role of TECs during the first weeks of the murine postnatal life is particularly challenging due to the significant augment in T cell production. Here, we critically review recent studies centered on the timely coordination between the expansion and maturation of TECs during this period and their specialized role in T cell development and selection. We further discuss how aging impacts on the pool of TEC progenitors and maintenance of functionally thymic epithelial microenvironments, and the implications of these chances in the capacity of the thymus to sustain regular thymopoiesis throughout life.

## Introduction

The current pandemic caused by the SARS-CoV-2 virus underscores the importance of maintaining a pool of immunologically competent T cells, which are capable of responding to virtually any new foreign threats while tolerant to the host own tissues. The establishment of a diverse T cell receptor (TCR) repertoire arises from the random recombination of V(D)J gene segments during T cell development in the thymus. Yet, the arbitrariness underlying this process can also produce autoreactive T lymphocytes. The thymus has developed several control mechanisms to simultaneously establish T cell immunity against non-self elements and impose self-tolerance. Particularly important in the choreography of T cell selection are thymic epithelial cells (TECs), which represent a key component of the thymic stromal microenvironment. TECs are typically subdivided into functionally distinct cortical (cTEC) and medullary (mTEC) lineages (1). While cTECs primarily mediate T cell lineage commitment and positive selection, mTECs fine-tune the negative selection of autoreactive thymocytes or promote their deviation into the T regulatory cell lineage (2). It is conceptually accepted that cTECs and mTECs differentiate from thymic epithelial progenitors (TEPs) present within the embryonic and postnatal thymus (2). Deficits in the function of TECs arise with aging, cytoablative regimens and infection, leading to a lower naïve T cell output. These thymic failures are pertinent in the elderly and patients undergoing bone marrow transplantations (BMT), contributing to their poor T-cell responses to new pathogens or predisposing to autoimmunity (3). Thus, the preservation of a regular thymic function also depends on the maintenance and differentiation potential of bipotent or lineage restricted TEPs. In this review, we focus on critical changes in the molecular traits of TECs that occur during the first weeks of the murine postnatal life, and integrate how these alterations might precede events coupled with thymic involution.

## The Build-Up of TEC Microenvironments

The initiation of TEC development coincides with the onset of thymus organogenesis, which starts around day 9-10 of the murine embryonic gestation (E9-10) (4). The expression of Forkhead box protein N1 (Foxn1) in the ventral area of the common thymus and parathyroid primordium marks a critical step in TEC specification (5). Still, Foxn1 expression needs to be continuously maintained during the differentiation of c/mTEC, wherein it imposes a complex genetic program that confers them the capacity to support distinct stages of thymopoiesis (6). TEPs formed during early thymus ontogeny constitute the primordial building blocks for the establishment and maintenance of c/mTEC microenvironments (7–9). Our comprehension about the mechanisms underlying TEC differentiation has considerably advanced with the identification of distinct populations containing bipotent or lineage-restricted progenitor activity (10–21) [further detailed below and reviewed in (22, 23)]. These studies led to the proposal of different refined models of TEC differentiation, whereby TEPs traverse through transitional stages that share a closer or distinct relationship with cTEC- or mTEC-unipotent precursors, prior to the commitment in mature c/mTEC subsets [reviewed in (2, 24, 25)]. Yet, it remains unclear the trajectories and molecular elements governing the differentiation of TEC progenitors into mature c/mTEC lineages.

The expansion and functionalization of c/mTEC compartment during early postnatal stages generates a supportive microenvironment that increases thymopoiesis, reaching its peak during young adulthood. Thereafter, T cell production progressively declines with aging, becoming residual in the aged thymus (26). During these periods, TECs undergo concomitant alterations in their composition and differentiation program. Although the density of TECs based on flow cytometry analysis might be underestimated (27), the number of TECs vigorously expands during postnatal life and early adulthood, followed by a progressive decline with age (28, 29). Changes in the size of TEC microenvironment appears to relate with the function of the thymus. While a reduction in the TEC compartment below a certain threshold restrains thymopoiesis (30, 31), the expansion of the thymic epithelial niche, for example *via* transgenic expression of Foxn1 or Cyclin D1, increases T cell generation (32, 33). Along this line, the frequency of cycling TECs is elevated during fetal life, progressively declines during the postnatal life and become a rare fraction in the aged mouse thymus (28). Transcriptomic analysis revealed that the expression of cell-cycle regulators is downregulated in TECs as early as 1 month (34). Moreover, the enforced expression of cMyc in TECs promotes the expansion of the TEC compartment, *via* the engagement of a genetic program akin to the one found in embryonic TECs (35). These results suggest that the loss in the proliferative rate of TECs, together with other alterations such as changes in cell survival and rate of differentiation, may contribute to a reduction in the size of TEC compartments with age. In the next sections, we outline specific cellular and molecular alterations that take place in c/mTEC during early postnatal life, and conjecture how those changes may anticipate subsequent functional losses in the capacity of TECs to sustain regular thymopoiesis in the long-term.

## The Assembly of Functionally Dedicated cTEC and mTEC Compartments

The first weeks of the postnatal life marks a period of intense turnover and functional diversification in the TEC niche, wherein key mature subsets in tolerance induction are generated or expanded (23). During this period, the changes in the cellularity and functionality of cTECs appear to unfold concomitant with the expansion and diversification of mTECs (11, 12, 36–38). This leads to a conspicuous inversion in the cTEC/mTEC ratio within the first 2 weeks after birth, which correlates with the intensification of thymopoiesis (11, 12, 28). In this regard, the consequent rise in the number of positive and negative selection events, will impose an increase demand on TEC compartments. Given that mature cTECs and mTECs have a limited life-span, the maintenance and specialization of their microenvironment seem to depend on the continual differentiation of their progenitors. These functional requirements are in part met by a symbiotic relationship with thymocytes (discussed further below) that stimulate specific proliferative and differentiation programs in TECs (39).

It remains surprising how little we know about the molecular program that underlies the differentiation of cTECs. Despite these gaps, several studies highlight that cTECs undergo molecular and functional changes during neonatal and puberty periods. In particular, cTECs downregulate the expression of key thymopoietic factors, such as Dll4 and IL-7, during the first weeks of postnatal life, which result from continual lymphoepithelial interactions (37, 38, 40, 41). These quantitative and qualitative disruptions in cTECs appear to anticipate the bona fide hallmarks that characterize TECs in the involuted thymus. In contrast to cTECs, our understanding of the cartography of mTEC differentiation is more complete (22). This process depends on reciprocal signals provided by several types of hematopoietic cells (1). These lymphoepithelial interactions, commonly referred as thymic crosstalk, engage specific members of the tumor necrosis factor receptor superfamily (TNFRSF), including receptor activator of NF-κB (RANK), CD40 and lymphotoxin β receptor (LTβR), in mTECs and their progenitors, leading to the activation of a nuclear factor kappa B (NF-κB)-dependent maturation program [reviewed in (1, 22)]. The cooperative action of TNFRSF members is not only important for the expansion of mTEC niches but also for their functional diversification. Upon the initial subdivision in mTEC^low^ and mTEC^high^ (42), the discovery of Autoimmune regulator (Aire)-, Ccl21- and forebrain embryonic zinc finger-like protein 2 (Fezf2)-expressing cells revealed that mTECs harbors a variety of functionally distinct mature subsets (1, 22). Although Aire^+^ and Fezf2^+^ cells emerge during embryonic life (1, 22), their abundance significantly increases in the first weeks of life. In this regard, RANK-mediated signaling is essential to the expansion of Aire^+^ mTECs, whereas CD40 also contributes to this process (43, 44). Although LTβR signaling was initially coupled to the development of Aire^+^ (45) and Fezf2^+^ lineages (46), subsequent studies indicated its involvement in the architecture of postnatal medullary compartment (47). Aire and Fezf2 regulate the capacity of mTECs to express large sets of non-overlapping tissue restricted antigens (TRAs), which are randomly organized in patterns of gene expression at the single cell level (48–50) and are reported to decrease their levels with age (51–53). In this regard, an earlier study underscore the importance of Aire expression in mTECs during neonatal period (54), which corelates with their capacity to control the generation of a unique population of T regulatory cells (55). It remains to be determined whether Aire expression during this temporal window particularly impacts on the quantity or quality of TRAs expression by mTECs.

The role of mTECs in tolerance induction extends beyond their promiscuous gene expression capacity. CCL21-producing cells represent a prototypical example of alternative roles of mTECs. CCL21-expressing mTEC represent a subset of mTEC^lo^ and control the migration of positively selected thymocytes towards the medulla (56, 57). CCl21^+^ cells emerge during embryogenesis and their numbers also undergo a marked increase during the first weeks of life (57). Recent single cell RNA sequencing analysis suggests that Aire- and Ccl21a-expressing mTEC subsets do not share a direct lineage relationship (58). Moreover, the discoveries that Aire^+^mTECs differentiate into Post-Aire cells (59, 60) further extended our view on the heterogeneity within thymus medulla. Post-Aire mTECs shutdown the expression of Aire, certain TRAs, CD80 and MHCII, while acquiring traits of terminally differentiated keratinocytes (61, 62). Two reports identified a highly differentiated mTECs that share molecular traits with tuft cells found at mucosal barriers. Fate-mapping analysis suggests that this subset can develop *via* an AIRE-dependent and AIRE-independent pathway (63, 64). Although their complete functional relevance remains elusive, tuft-like mTECs appear to regulate the development of invariant NKT cells and ILCs (63, 64). Future studies may uncover new specialized mTEC subsets and their role in imposing the limits of tolerance, or alternative processes in thymus biology.

## The Thymic Epithelial Cell Progenitor Reservoir

The diversification of TECs during the first weeks of life is dictated by the intricate balance between the rate of proliferation and differentiation of mature subsets. The rapid turnover of TEC microenvironments, with an estimated replacement time of one to two weeks to mTECs (28, 59), implicates the requirement for a regular generation of mature TECs from their upstream progenitors. One possibility is that bipotent TEPs continually produce lineage-committed precursors lacking long-term self-renewal capacity. Alternatively, and not mutually exclusive, the abundance of bipotent TEPs might decrease with age, being the maintenance of cortical and medullary epithelial niches assured by downstream compartment-restricted precursors. In the last years, several studies provide evidence for the existence of an arsenal of subsets enriched in purported bipotent TEC progenitors in the postnatal thymus (10, 13–15). One approach has employed *in vitro* 2D-clonogenic (10) or spheroids (13) assays to respectively isolate TEC progenitors that reside within EpCAM^+^Ly51^+^cTECs or EpCAM^-^ cells, which were expanded *in vitro* and revealed the capacity to give rise to c/mTEC. Nonetheless, a more recent study indicate that cells isolated from EpCAM^-^derived spheroids represent mesenchymal progenitors (65). Other methodologies resolved bipotent progenitor activity within defined subsets of UEA-1^−^MHII^lo^Sca-1^+^ TECs (14) and MHCII^hi^ Ly51^+^Plet1^+^ cTECs (15). Both strategies employ reaggregate organ cultures (RTOCS) to determine the precursor-product lineage relationship to mature cells. Despite the advances, it remains to be determined the physiological contribution of these cells to the TEC microenvironment in the adult thymus. Thus, we still lack experimental evidence that demonstrates the existence of bona-fide bipotent TEC progenitors in the postnatal thymus, and their identification at the single cell level.

Downstream of TEC progenitors, complementary studies documented how mTEC compartments evolved from bipotent TEP and mTEC-restricted precursors (mTEPs), including mTEC-restricted SSEA-1+ and podoplanin+ (PDPN) mTEPs (16, 18). Fate-mapping studies show that the adult mTEC network arise from fetal- and newborn-derived TEPs expressing beta5t (β5t), a prototypical cTEC marker. Yet, the contribution of β5t+ TEPs to the adult mTEC niche decreases with age (19, 20), suggesting that the maintenance of the adult medullary epithelium is assured by mTEPs. Although bipotent TEPs might lose the expression of some traits found in the embryo (e.g. β5t), it is also possible that the abundance and/or the self-renewal properties of bipotent TEPs and/or lineage-restricted progenitors decline with time. Supporting this view, the clonogenic activity of purported bipotent TEPs that reside within the cortex decrease with age (10) and Cld3,4^+^SSEA1^+^ mTEC-restricted cells become rare in the adult thymus (16). Given that the numbers of embryonic TEPs dictates the size of functional TEC microenvironments (30), we infer that the loss in the TEC network that takes place with age may result from the decrease in the bioavailability and self-renewal capacity of TEPs early in life.

The advent of single cell RNA sequencing (scRNAseq) analysis have also contributed to our understanding of the heterogeneity and dynamic of TEC progenitors. This approach has emerged as a new unbiased method to identify novel subsets, providing a valuable platform to analyze their developmental trajectories and determine their relationships with progenitor subsets identified by conventional methodologies. In this regard, new clusters termed “pre-Aire mTEC 1 and 2” (66) appear to present molecular traits similar to the ones found in podoplanin+ (PDPN) mTEPs (18). A subsequent study identified a novel cluster of “intertypical TECs” (51) that harbors traits akin to the ones found in podoplanin+ (PDPN) mTEPs (18), UEA-1^−^MHII^lo^Sca-1^+^ (14) and MHCII^hi^ Ly51^+^Plet1^+^ (15) TECs. Since “intertypical TECs” are further segmented in distinct 4 subclusters, it would be interesting to determine if they associate to a particular bipotent or unipotent subset. Moreover, scRNAseq analysis reveal the existence of a previously unrecognized cluster of “perinatal cTECs”. Interestingly, this subset harbors cells with a highly proliferative status and their abundance declines with age (51). Moreover, the combination of scRNAseq and fate mapping analysis revealed that β5t^+^ TEPs acquire senescent-like properties with age, potentially explaining their failure to contribute to mTEC lineage beyond the neonatal stage (19, 20). Together, these findings indicate that the integration of multiple experimental approaches provides a more complete strategy to resolve the intricacies of the TEC compartment. Future studies should attempt to identify specific markers to resolve the newly characterized populations at a single level.

## Concluding Remarks

The aforementioned studies underscore that the period between birth and early adulthood is a time of intense alterations in TEC microenvironments, which prepares them to the highly demand role of choreographing the selection of growing number of T cell precursors. In this sense, it is remarkable to appreciate the synchronous coordination between TEC differentiation and the requisites imposed by T cell development. Yet, the erosion of the pool of TEC progenitors seem to accompany the generation of specialized subsets with key roles in tolerance induction. We reason that an in-depth molecular analysis of TEC differentiation during early postnatal may provide insights on how TEC niches are maintained, and can be repaired in the aged thymus. Despite recent advances, it remains unclear how changes in the bioavailability of TEPs impact on the maintenance of TEC microenvironment across life, and ultimately on thymic output. Another unexplored area pertains to the physiological causes underlying the presumed age-dependent decrease and/or senescence of TEPs. Knowledge in these areas will not only permit to comprehend the basic principles that governs thymic function, but also target pathways for the treatment of disorders coupled to dysfunctional thymic/T cell responses.

## Author Contributions

NA and RP wrote the manuscript. All authors contributed to the article and approved the submitted version.

## Funding

This work was supported by a starting grant from the European Research Council (ERC) under the project 637843 and by FEDER - Fundo Europeu de Desenvolvimento Regional funds through the COMPETE 2020 - Operacional Programme for Competitiveness and Internationalisation (POCI), Portugal 2020, and by Portuguese funds through FCT - Fundação para a Ciência e a Tecnologia/Ministério da Ciência, Tecnologia e Ensino Superior in the framework of the project POCI-01-0145-FEDER-029129 (PTDC/MED-IMU/29129/2017) and PTDC/MED-IMU/1416/2020. NA is supported by the FCT program “Scientific Employment Stimulus” and RP by a FCT PhD fellowship.

## Conflict of Interest

The authors declare that the research was conducted in the absence of any commercial or financial relationships that could be construed as a potential conflict of interest.
